# Genetic dissection of Septoria tritici blotch and Septoria nodorum blotch resistance in wheat using GWAS

**DOI:** 10.3389/fpls.2025.1524912

**Published:** 2025-05-13

**Authors:** Alma Kokhmetova, Nagenahalli Dharmegowda Rathan, Deepmala Sehgal, Shaukat Ali, Yuliya Zeleneva, Madina Kumarbayeva, Ardak Bolatbekova, Gopalareddy Krishnappa, Zhenis Keishilov, Asia Kokhmetova, Kanat Mukhametzhanov, Kanat Bakhytuly

**Affiliations:** ^1^ Laboratory of Breeding and Genetics, Institute of Plant Biology and Biotechnology (IPBB), Almaty, Kazakhstan; ^2^ Discovery Breeding Team, Corteva Agriscience, Hyderabad, Telangana, India; ^3^ Syngenta, Jealott’s Hill International Research Centre, Bracknell, United Kingdom; ^4^ Agronomy, Horticulture, and Plant Science Department, South Dakota State University, Brookings, SD, United States; ^5^ Laboratory of Mycology and Phytopathology, All Russian Institute of Plant Protection, St. Petersburg-Pushkin, Russia; ^6^ Department of Genetics and Plant Breeding, Indian Council of Agricultural Research (ICAR)-Sugarcane Breeding Institute, Coimbatore, India

**Keywords:** MTAs, Septoria tritici blotch, Septoria nodorum blotch, GWAS, candidate genes

## Abstract

**Introduction:**

Septoria blotch is a globally significant disease, which ranks second in importance after wheat rusts that causes substantial yield losses. The development of Septoria blotch resistant cultivars through molecular approaches is both economical and sustainable strategy to contain the disease.

**Methods:**

For identifying genomic regions associated with resistance to Septoria tritici blotch (STB) and Septoria nodorum blotch (SNB) in wheat, a genome-wide association study (GWAS) was conducted using a diverse panel of 191 spring and winter wheat genotypes. The panel was genotyped using DArTseq™ technology and phenotyped under natural field conditions for three cropping seasons (2019–2020, 2020–2021, and 2021–2022) and under artificially inoculated field conditions for two cropping seasons (2020–2021 and 2021–2022). Additionally, the panel was phenotyped under greenhouse conditions for STB (five mixed isolates in a single experiment) and SNB (four independent isolates and a purified toxin in five different independent experiments).

**Results and Discussion:**

GWAS identified nine marker–trait associations (MTAs), including six MTAs for different isolates under greenhouse conditions, two MTAs under natural field conditions, and one MTA under artificially inoculated field conditions. A pleiotropic MTA (100023665) was identified on chromosome 5B governing resistance against SNB isolate Pn Sn2K_USA and SNB purified toxin Pn ToxA_USA and explaining 30.73% and 46.94% of phenotypic variation, respectively. *In silico* analysis identified important candidate genes belonging to the leucine-rich repeat (LRR) domain superfamily, zinc finger GRF-type transcription factors, potassium transporters, nucleotide-binding site (NBS) domain superfamily, disease resistance protein, P-loop containing nucleoside triphosphate hydrolase, virus X resistance protein, and NB-ARC domains. The stable and major MTAs associated with disease resistant putative candidate genes are valuable for further validation and subsequent application in wheat septoria blotch resistance breeding.

## 1 Introduction

Wheat (*Triticum aestivum* L.) plays an important role in global food and nutritional security, providing 20% of the world’s calories and proteins (FAO, 2023). Being a staple food for 40% of the world’s population, it is a critical part of the daily diet in many regions. The demand for food products derived from wheat has increased due to population growth, changing dietary patterns, and rising income levels in the era of urbanization (Krishnappa et al., 2023; Pardey et al., 2014). To ensure the food security of the fast-growing world population, the average annual yield should increase from 1.2% to 1.6% (Khan et al., 2023; Krishnappa et al., 2021). Significant research efforts are also required to protect wheat from biotic and abiotic stresses (Khan et al., 2024; Reynolds et al., 2007), particularly when the new pathogen races are adopted in non-conventional areas (Kokhmetova et al., 2023).

Septoria tritici blotch (STB), caused by a hemibiotrophic fungus, *Zymoseptoria tritici*, is a big threat to wheat production worldwide that can cause yield losses between 35% and 50% (Patial et al., 2024). The septoria nodorum blotch (SNB), caused by the necrotrophic fungus *Parastagonospora nodorum*, causes yield losses between 20% and 50% (Simón et al., 2002). The septoria blotch disease ranks second in importance after wheat rusts in the United States and number one in Russia and many Western European nations (Ponomarenko et al., 2011; Koyshibaev, 2018). Wheat production in Kazakhstan is also highly affected by septoria epidemics. In northern Kazakhstan, disease outbreaks occur approximately five times every decade (Koyshibaev, 2018). Understanding the gene-for-gene interactions in the *P. nodorum–*wheat system facilitates more effective resistance breeding (Friesen and Faris, 2021). In this interaction, wheat host sensitivity genes recognize *P. nodorum* necrotrophic effectors (NEs) that promote disease by inducing host hypersensitivity and programmed cell death (Oliver et al., 2012). Owing to *P. nodorum* being a necrotroph, this recognition results in the pathogen gaining nutrients from the dying tissue, which allows the disease to progress. To date, nine host gene and pathogen effector interactions were characterized, namely, *Tsn1*–SnToxA, *Snn1*–SnTox1, *Snn2*–SnTox267, *Snn3-B1*–SnTox3, *Snn3-D1*–SnTox3, *Snn4*–SnTox4, *Snn5*–SnTox5, *Snn6*–SnTox267, and *Snn7*–SnTox267. The genes for five effectors (*SnTox1*, *SnTox3*, *SnToxA*, *SnTox5*, and *SnTox267*) and three host genes (*Tsn1, Snn1*, and *Snn3-D1*) were cloned (Peters Haugrud et al., 2022). Each NE interacts with host sensitivity genes (*Tsn1, Snn1, Snn2, Snn3, Snn4, Snn5, Snn6*, and *Snn7*) (Friesen et al., 2009). The cloned susceptibility genes belong to distinctly different classes, which include an intracellular protein featuring protein kinase, nucleotide-binding, and leucine-rich repeat (LRR) domains (*Tsn1*), a wall-associated kinase (*Snn1*), and a protein kinase related to major sperm proteins (*Snn3-D1*) (Faris et al., 2010; Shi et al., 2016; Zhang et al., 2021).

The development of genetically resistant cultivars using marker-assisted breeding is the ideal approach to mitigate the effects of this pathogen (Siah et al., 2014). For example, a lot of qualitative genes conferring resistance to STB at different growth stages were identified. To date, 23 major genes, namely, *Stb1* to *Stb20*, *StbSm3*, *StbWW*, and *TmStb1*, have been identified on different chromosomes including two cloned genes, i.e., *Stb6* and *Stb18q*, encoding a wall-associated receptor kinase-like protein and a plasma membrane cysteine-rich receptor-like kinase, respectively (Dreisigacker et al., 2015; Patial et al., 2024). In addition, several QTLs associated with resistance to STB have been identified in wheat on multiple linkage groups, highlighting the importance of variation and the complex genetics of this disease (Brown et al., 2015; Louriki et al., 2021). However, absolute resistance is currently not available for septoria blotch, and the resistance is further governed by many genes encoding different disease resistance traits. Also, the QTLs identified using biparental mapping extend to several megabases physically on the reference genome, making the identification of candidate genes an arduous task.

The two commonly used methods to dissect complex quantitative traits are QTL mapping and genome-wide association study (GWAS). Genetic dissection of disease resistance through GWAS can profoundly improve the power of QTL identification by significantly increasing the mapping resolution in comparison with bi-parental-based QTL mapping, since it accounts historical recombination events, high genetic diversity, and high polymorphism detected by markers in a germplasm panel. Many high-throughput genotyping platforms have become available in wheat, which have made GWAS possible for a plethora of traits in this polyploid species including resistance to Septoria blotch disease (Kollers et al., 2013; Miedaner et al., 2013; Adhikari et al., 2015; Dreisigacker et al., 2015; Mirdita et al., 2015; Kidane et al., 2017; Muqaddasi et al., 2019; Odilbekov et al., 2019; Alemu et al., 2021; Rathan et al., 2022: Khan et al., 2022; Krishnappa et al., 2022; Khan et al., 2024; Sehgal et al., 2024). The present study aimed to explore the bread wheat panel, assembled for GWAS as part of the CIMMYT-ICARDA-IWWIP (International Maize and Wheat Improvement Center-International Center for Agricultural Research in the Dry Areas–International Winter Wheat Improvement Program) partnership program, for identifying genomic regions contributing resistance to STB and SNB in wheat. The same panel was successfully used earlier for mapping tan spot resistance (Kokhmetova et al., 2021). The present study has furthered our understanding of the genetic architecture of Septoria blotch resistance in wheat.

## 2 Materials and methods

### 2.1 Experimental materials

The GWAS population is composed of 191 wheat genotypes including 89 spring wheat and 102 winter wheat genotypes that consisted of 111 cultivars and breeding lines from Kazakhstan, 17 cultivars from Russia, and 1 cultivar from Brazil, as well as 30 lines sourced from CIMMYT and CIMMYT-ICARDA-IWWIP. Most importantly, the cultivars in the panel are extensively used in breeding programs in Kazakhstan and Central Asian countries.

### 2.2 Inoculum production, inoculations, and seedling test in greenhouse

The GWAS panel was phenotyped under greenhouse conditions for *Z. tritici* (mix of five isolates, namely, 156-22, 154-22, 1-22, 170 6-22, and 3-22 screened in a single experiment) and *P. nodorum* (four independent isolates, namely, 149-22_ToxA, 150-22_Tox1, and 118-22_Tox3 from Russia and Sn2K from USA and a purified toxin SnToxA from USA screened in five different independent experiments) during 2023. The *P. nodorum* isolates 149-22_ToxA, 150-22_Tox1, and 118-22_Tox3, originating from the Tambov and Altay regions of Russia, were identified as producers of toxins ToxA, Tox1, and Tox3, respectively. This identification was confirmed by inoculation experiments with differential wheat genotypes (“Mironovskaya 808” as a susceptible check, “Don Mira” as a resistant check) infiltration assays, and PCR techniques using ToxA-, Tox1-, and Tox3-specific markers (Nuzhnaya et al., 2023; Kovalenko et al., 2023; Zeleneva et al., 2023). All isolates were received from the All-Russian Institute of Conservation, Russia, except isolate Sn2K and purified toxin SnToxA, which are from South Dakota State University (SDSU), USA. Hence, six independent experiments were conducted in greenhouse conditions. The experiments were conducted at the All-Russian Institute of Plant Protection (ARIPP) in St. Petersburg-Pushkin, Russia. However, the experiments using isolate Sn2K and purified toxin SnToxA were conducted at SDSU in Brookings, SD, USA. The wheat genotypes were grown in a completely randomized design with five replications. Ten seeds from each genotype were sown in 20-cm pots, with each pot serving as one replicate. Soil preparation and inoculation followed standard protocols, using a universal substrate (“Terra vita” produced by “Nord Pflp,” Russia). The data generated from greenhouse experiments are given in **Supplementary Table S1**.

The *P. nodorum* isolates were preserved on V8-PDA agar at 21°C in a 12-h light and dark cycle for 2 weeks (Phan et al., 2016). Stock cultures of *Z. tritici* were grown on yeast sucrose agar (YSA; 10 g L^−1^ yeast extract, 10 g L^−1^ sucrose, and 1.2% agar) with kanamycin (50 µg/mL) supplement (Scala et al., 2020). Thirty-day-old cultures were stored in a refrigerator at +4°C temperature for inoculation prior to use (Scala et al., 2020). Inoculation with foliar pathogens involved spraying conidial suspensions (*P. nodorum*: 10^6^ spores/mL; *Z. tritici*: 10^7^ spores/mL) containing 0.1% Tween 20 surfactant, as described by Scala et al. (2020) and Fagundes et al. (2020). The inoculum was evenly sprayed on the plants, and the pots containing plants were kept in the climate chamber (Model MLR-352H-PE, “PHCbi”, Tokyo, Japan). A thoroughly cleaned spray gun was set at 2.0 bar pressure to spray the *Z. tritici*/*P. nodorum* inoculum on the selected marked leaf sections of each plant. Approximately 15 min were allowed to settle the inoculum on the leaf surface. The pots were then kept in big plastic bags containing approximately a liter of water. To create a congenial environment of high relative humidity (RH), the bags were tightly closed with tape or plastic clips. Furthermore, a greenhouse facility was used to incubate the plants containing bags at approximately 20°C during the day and ~12°C at night with a 12-h day/12-h night cycle. After a 48-h treatment cycle, the pots were taken out from the bags and kept in the trays, ensuring randomized placement. To maintain the RH of 70% to 90%, the plants were given water at frequent intervals. The same growth conditions including light and temperature were used for the wheat plants to grow for 21 days after inoculation (Kolomiets et al., 2017; Fagundes et al., 2020).

The disease reaction to *P. nodorum* isolates from Russia was assessed at 20–22 days post-inoculation through a lesion-based scale of 0–4, where 0–1 indicates resistance, 2 indicates moderate susceptibility, 3 indicates susceptibility, and 4 indicates high susceptibility (Tan et al., 2012). For the screening of wheat with the *P. nodorum* isolate Sn2K from the USA, inoculation was performed using conidia as described by Liu et al. (2004). Additionally, for phenotyping sensitivity to SnToxA, lines were screened for their reaction to the purified toxin Ptr ToxA, which is equivalent to SnToxA, at a concentration of 10 µg/mL. Four leaves from each genotype (with the second leaf fully expanded) were infiltrated with pure SnToxA culture filtrate as detailed in Faris et al. (1996). Post-infiltration, the plants were kept at 21°C during the day and 18°C at night, with a 16-h photoperiod in the growth chamber. Leaves were scored as insensitive (−) or sensitive (+) after 4 days of infiltration.

Now, on *P. nodorum* isolates, 149-22_ToxA, 150-22_Tox1, 118-22_Tox3, Sn2K isolate, and SnToxA will be quoted as Pn ToxA_Russia, Pn Tox1_Russia, Pn Tox3_Russia, Pn Sn2K_USA, and Pn ToxA_USA, respectively.

### 2.3 Field phenotyping

The phenotyping data of three cropping seasons under natural field conditions and two cropping seasons under artificial field inoculation conditions are given in **Supplementary Table S2**. The GWAS population was evaluated at the Kazakh Research Institute of Agriculture and Plant Growing (KRIAPG), Almalybak (43°1300900 N, 76°3601700 E) in Southeast Kazakhstan, Almaty, during the 2019–2020, 2020–2021, and 2021–2022 cropping seasons with a plot size of 1 m^2^. The experimental material was given a fertilizer dose of 60 kg/ha N and 30 kg/ha P_2_O_5_ and standard crop management practices were followed (Dospekhov, 1985). The planting material was sown during mid-September and was harvested in mid-August of the succeeding year during all three years of the testing period. The region receives 400 mm of rainfall annually; hence, only three irrigations were given during crop growth. The field phenotyping was done under natural field disease incidence conditions for three consecutive years during 2019–2020, 2020–2021, and 2021–2022 crop seasons, whereas phenotyping was done under artificial field inoculation conditions for two consecutive years i.e., 2020–2021 and 2021–2022. Field plots were inoculated with a mixed inoculum of *Z. tritici*, derived from 80 to 100 randomly selected infected leaf samples collected from major spring wheat-producing regions in southeastern Kazakhstan. The diseased straw and stubbles were added to the soil at the rate of 1 kg/m^2^ before sowing.

The Zadoks scale was used to score the disease incidence in the field; the disease severity was scored on the first and flag leaves when all the lines were near or at Zadoks growth stage Z69 (complete flowering stage) and Z75 (medium milking stage) (Zadoks et al., 1974). The STB score was determined by calculating the percentage of infection on individual leaves and averaging the multiple scorings. A double-digit scale of 00–99, which was modified from Saari and Prescot (Saari and Prescott, 1975), was used to classify host reactions to STB. Based on the degree of infection, the genotypes were divided into the following categories: 0%–10% rated as highly resistant (HR: infection-free or some scattered lesions on the lower leaves); 11%–20% rated as resistant (R: low-intensity infection on first leaves and isolated lesions on the second set of leaves); 21%–40% rated as moderately susceptible (MS: lower leaves’ infection is mild to severe and isolated to the low infection spreading to the leaf below the mid portion of plant); 41%–70% rated as susceptible (S: high-intensity lesions on leaves present at the low and middle portion of the plant and mild to high infection of the upper third of plant; flag leaf infection is higher than the traces); 71%–100% rated as highly susceptible (HS: infections spread to spikes and very high infections on all the leaves). The phenotype ratings for STB resistance were calculated as an area under the disease progress curve (AUDPC).

### 2.4 Phenotypic data analysis

The field (natural infection and artificial infection) resistance for STB was calculated as AUDPC scores. The AUDPC was estimated yearly by cumulating the progress of diseases severity. AUDPC values from double-digit and AUDPC from flag leaf (F) and penultimate leaf (F−1) were separately estimated using the formula defined by Wilcoxson et al. (1974). The AUDPC was calculated based on three STB severity scores taken at 7-day intervals during plant growth. The formula used to calculate the AUDPC was as follows:


$$AUDPC=\;\sum_{i=1}^{n-1}\frac{y_{i}+\;y_{i+1}}{2}\times \left(t_{i+1}-\;t_{i}\right)$$

*y_i_* is an evaluation of disease at the *i*th observation;

*t_i_* is time (in days) at the *i*th observation;

*n* is the total number of observations.

All phenotypic analysis was done in multi-environment trial analysis in R (META-R) version 6.0 (Alvarado et al., 2015). In brief, the single year Best Linear Unbiased Estimators (BLUEs) were estimated for the greenhouse evaluations for isolates from *Z. tritici* and *P. nodorum*, namely, Zt_Russia (mixed isolates), Pn ToxA_Russia, Pn Tox1_Russia, Pn Tox3_Russia, Pn Sn2K_USA, and Pn ToxA_USA. While calculating BLUEs, the genotypes are considered as fixed effects. Furthermore, genetic and residual variances, broad sense heritability (H2), coefficient of variance (CV), mean, and correlation coefficients were estimated in META-R. These generated AUDPC scores from field evaluations and BLUEs from greenhouse evaluations were used for GWAS analysis.

### 2.5 DNA extraction and genotyping

The details of DNA extraction and genotyping is provided in Kokhmetova et al. (2021). Briefly, DNA was extracted from fresh young leaves following the modified CTAB method as described in Dreisigacker et al. (2016). The genotyping was done with DArTseqTM technology by the Genetic Analysis and Service for Agriculture (SAGA) laboratory in Mexico. Furthermore, the DNA libraries were sequenced with 192-plexing on Illumina HiSeq2500 with 1 × 77-bp reads. The allele calls were generated by a proprietary analytical pipeline developed by DArT P/L (Sansaloni et al., 2011). The monomorphic markers, markers with minor allele frequency less than 5%, markers with > 20% missing allele calls, and markers with > 25% heterozygote frequency were removed to get the high-quality informative markers. The final filtered set of 8,154 markers were further used in GWAS analysis for the marker–trait association (MTA) identification. The genotyping data for 191 wheat entries are given in **Supplementary Table S3**.

### 2.6 Linkage disequilibrium and population structure

The estimation of pairwise linkage disequilibrium (LD) values (*r* ^2^) between the SNPs, construction of LD decay plots, Principal component analysis (PCA), and construction of Neighbor – Joining (N-J) tree for understanding population structure is done as described in Rathan et al., 2022. Briefly, the LD values (*r* ^2^) were generated using TASSEL version 5.2.94 and the LD decay was visualized in R Studio by following the method given by Remington et al. (2001). The point where the LD values drop to half of their maximum value is used to define the extent of LD decay at the genome and subgenome level. The distance matrix was generated in TASSEL version 5.2.94 and exported in Newick format, and the same matrix was used to generate N-J tree in iTOL version 7 tool (iTOL: Interactive Tree Of Life). The PCA was done using Genome Association and Prediction Integrated Tool (GAPIT) version 3.4.

### 2.7 Genome wide association studies and *in silico* analysis

The information about the procedures followed to perform GWAS analysis, generate QQ plots, and fix the Bonferroni correction factor and *R* ^2^ values is provided in Khan et al. (2022). Briefly, AUDPC scores from field evaluations and BLUEs from greenhouse evaluations were used for GWAS analysis. The BLINK (Bayesian-information and Linkage-disequilibrium Iteratively Nested Keyway) model (Huang et al., 2019) from GAPIT version 3.0 (Wang and Zhang, 2021) was employed to identify MTAs. The Bonferroni correction has been employed to adjust the threshold for statistical significance (α) at 0.05 and subsequently dividing this value by the total number of markers under consideration. The *R* ^2^ was used to describe the percentage variation explained (PVE) by significant MTAs. The allelic difference of significant MTA was estimated as the difference between the mean value of genotypes with and without favorable alleles for disease scores and was presented in box plots.

The *in silico* analysis was done as described in our previous study (Kokhmetova et al., 2023). In brief, the putative candidate genes were identified in RefSeq v2.1 assembly from the International Wheat Genome Sequencing Consortium (IWGSC) integrated in the Ensembl Plant database (https://plants.ensembl.org/index.html) using the basic local alignment search tool (BLAST). The 100-kb region overlapping and flanking the associated SNP was mined to identify the putative candidate genes.

## 3 Results

### 3.1 Phenotypic summary statistics and trait correlations

The BLUEs generated for different Septoria isolates tested under greenhouse conditions during 2023 and the summary statistics from the study are presented in **Table 1**. Heritability was high for all the studied isolates, ranging from 0.90 to 0.98. The genetic variance was highly significant for all phenotypes (**Table 1**). The coefficient of variation (CV) ranged from 12.89 (Pn ToxA_Russia) to 28.5 (Pn ToxA_USA). Genotypic and phenotypic correlation coefficients for various Septoria reactions tested under greenhouse conditions are presented in **Table 2**. The reaction of Zt_Russia mixed isolates had a significant and positive genetic correlation with isolates Pn ToxA_Russia (0.16*), Pn Tox1_Russia (0.3**), and Pn Sn2K_USA (0.28**). Pn ToxA_Russia isolate reaction had a significant and positive genetic correlation with Pn Tox1_Russia (0.35**), Pn Tox3_Russia (0.15*), and toxin Pn ToxA_USA (0.15*). The Pn Tox3_Russia isolate had a significant and positive genetic correlation with the Pn Sn2K_USA (0.15*) isolate. The Pn Sn2K_USA isolate had a significant and positive genetic correlation with the reaction to the toxin Pn ToxA_USA (0.69**). Similarly, Zt_Russia mixed isolates had a significant and positive phenotypic correlation with isolates Pn ToxA_Russia (0.14*) and Pn Tox1_Russia (0.29**). The Pn ToxA_Russia isolate has a significant and positive phenotypic correlation with Pn Tox1_Russia (0.34**), Pn Tox3_Russia 0.15*), and Pn ToxA_USA (0.16*). Since trait heritability is high, the phenotypic and genotypic correlations were very close to each other.

**Table 1:** Genetic parameters from 191 wheat accessions screened under greenhouse conditions during 2023 for STB and SNB.

Statistics	Zt_Russia	Pn ToxA_Russia	Pn Tox1_Russia	Pn Tox3_Russia	Pn Sn2K_USA	Pn ToxA_USA
Heritability	0.92	0.98	0.98	0.91	0.97	0.9
Genetic variance	0.45	0.77	0.86	0.43	1.38	0.62
Residual variance	0.2	0.09	0.1	0.2	0.2	0.35
Grand mean	2.33	2.28	2.15	2.4	2.64	2.06
LSD	0.55	0.37	0.39	0.56	0.72	0.73
CV	19.01	12.89	14.51	18.72	17.04	28.5
Genetic significance	2.2E-143	2.0E-318	3.6E-317	4.2E-137	1.1E-130	1.3E-119

LSD, least significant difference; CV, coefficient of variance; Zt_Russia, *Z. tritici* mixed isolates from Russia; Pn ToxA_Russia, *P. nodorum* isolate 149-22_ToxA from Russia; Pn Tox1_Russia, *P. nodorum* isolate 150-22_Tox1 from Russia; Pn Tox3_Russia, *P. nodorum* isolate 118-22_Tox3 from Russia; Pn Sn2K_USA, *P. nodorum* isolate Sn2K from USA; and Pn ToxA_USA, *P. nodorum* toxin SnToxA from USA.

**Table 2:** Correlation coefficients between different septoria isolates screened on 191 wheat genotypes in greenhouse.

Traits	Zt_Russia	Pn ToxA_Russia	Pn Tox1_Russia	Pn Tox3_Russia	Pn Sn2K_USA	Pn ToxA_USA
Zt_Russia	1.00	0.16*	0.3**	0.05	0.28**	−0.03
Pn ToxA_Russia	0.14*	1.00	0.35**	0.15*	0.09	0.15*
Pn Tox1_Russia	0.29**	0.34**	1.00	−0.02	0.08	−0.04
Pn Tox3_Russia	0.05	0.15*	−0.01	1.00	0.15*	0.07
Pn Sn2K_USA	0.10	0.05	0.01	0.12	1.00	0.69**
Pn ToxA_USA	−0.01	0.16*	−0.03	0.06	0.58	1.00

Zt_Russia, *Z. tritici* mixed isolates from Russia; Pn ToxA_Russia, *P. nodorum* isolate 149-22_ToxA from Russia, Pn Tox1_Russia, *P. nodorum* isolate 150-22_Tox1 from Russia; Pn Tox3_Russia, *P. nodorum* isolate 118-22_Tox3 from Russia; Pn Sn2K_USA, *P. nodorum* isolate Sn2K from USA; and Pn ToxA_USA, *P. nodorum* toxin SnToxA from USA.

The lower diagonal indicates phenotypic and the upper diagonal indicates genotypic correlation coefficients. **Significant at the 0.01 significance level, *Significant at the 0.05 significance level.

### 3.2 Marker’s statistics

The GWAS analysis was conducted with 8,154 high-quality SNP markers. The subgenome and chromosome level distribution is provided in **Table 3**. A, B, and D subgenomes had 3,298, 3,941, and 915 markers, respectively. At the chromosome level, the 4D chromosome harbored only 28 markers, whereas chromosome 2B harbored the maximum number of 870 markers.

**Table 3:** Subgenome and chromosome level distribution of markers.

Subgenome	Chromosome							Total
	1	2	3	4	5	6	7	
A	464	579	469	367	442	412	565	3,298
B	608	870	662	202	769	476	354	3,941
D	167	295	129	28	79	109	108	915
Total								8,154

### 3.3 Principal component analysis and linkage disequilibrium

**Figure 1:**
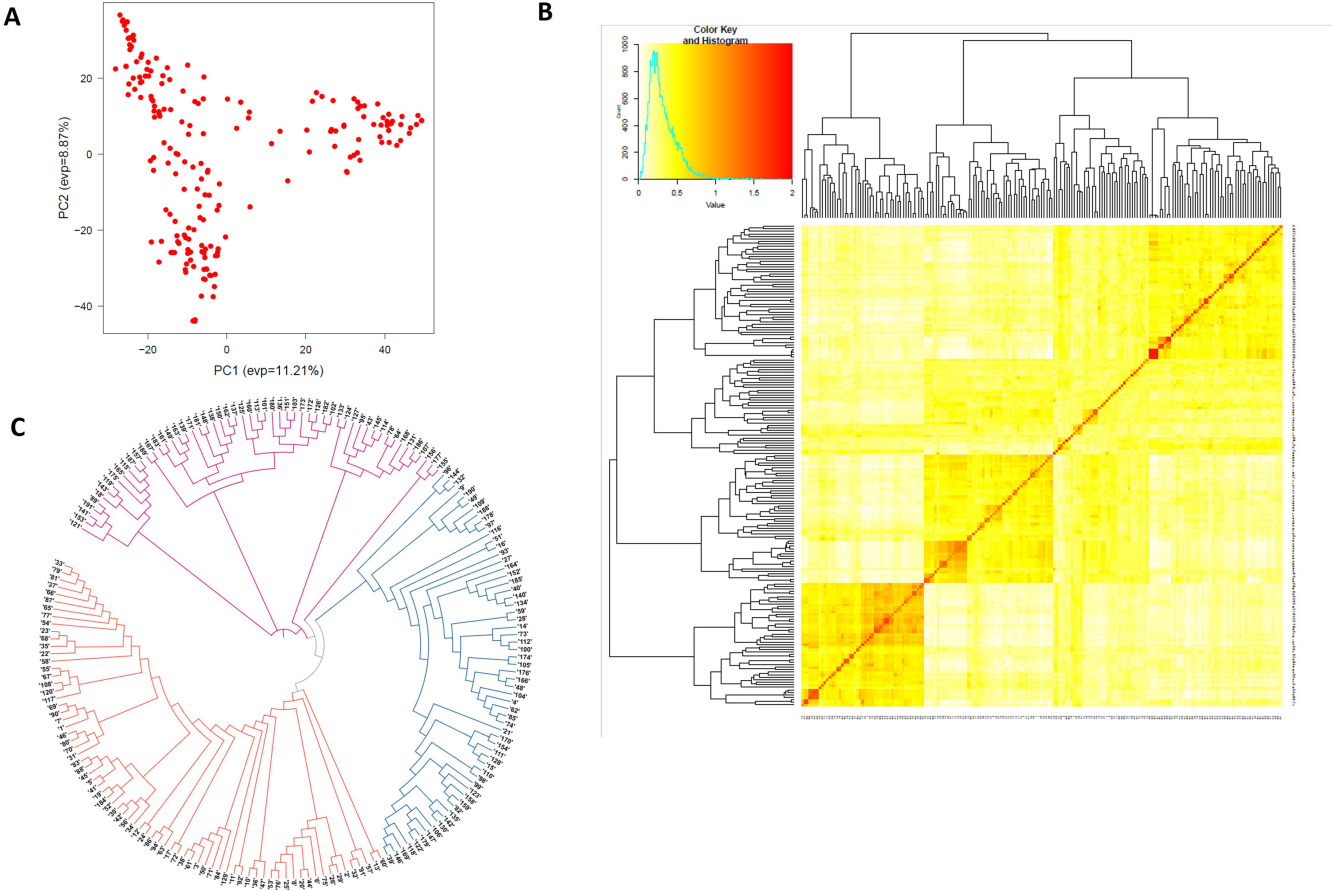
Structure analysis showing three genetic clusters among 191 wheat accessions in the GWAS panel. **(A)** Population structure based on principal component analysis, **(B)** heat map of pairwise kinship matrix, and **(C)** Neighbor-Joining (NJ) tree.

**Figure 2:**
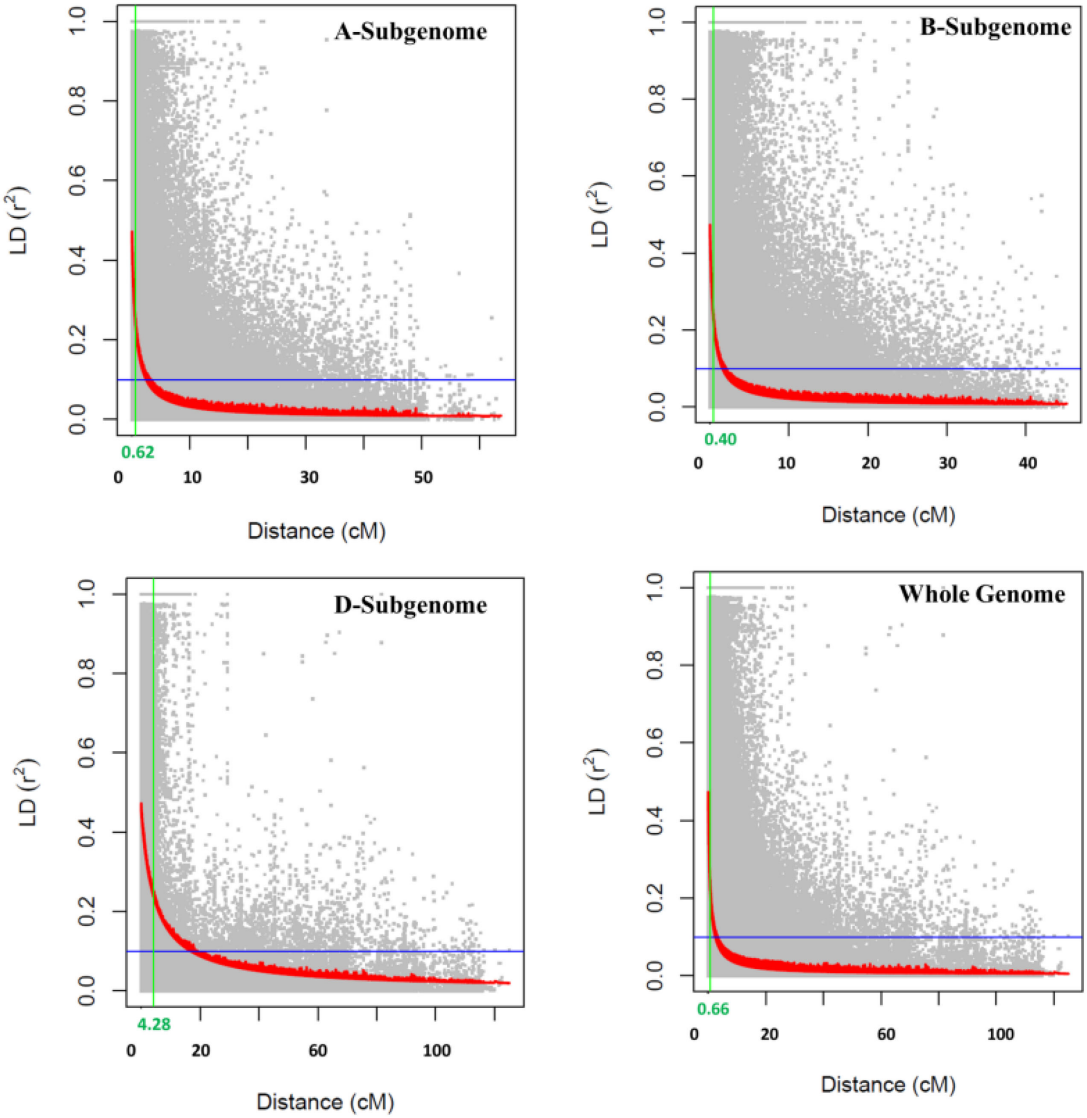
Genome- and subgenome-wise LD decay in the GWAS panel consisting of 191 wheat genotypes.

The PCA plot-based population structure is presented in **Figure 1A**. The heat map of the pairwise kinship matrix is presented in **Figure 1B**. Through Neighbor-Joining (NJ) analysis, the population was divided into three subgroups (**Figure 1C**). The *r* ^2^ values for all the SNPs were estimated and plotted against the genetic distance (cM) to calculate the LD values (**Figure 2**). The LD decay was rapid in the B subgenome (0.40 cM) followed by the A subgenome (0.62 cM) and the whole genome (0.66 cM). However, LD decay was much slower in the D subgenome (4.28 cM) as compared to the A and B subgenomes.

### 3.4 GWAS analysis

#### 3.4.1 Greenhouse experiments

**Figure 3:**
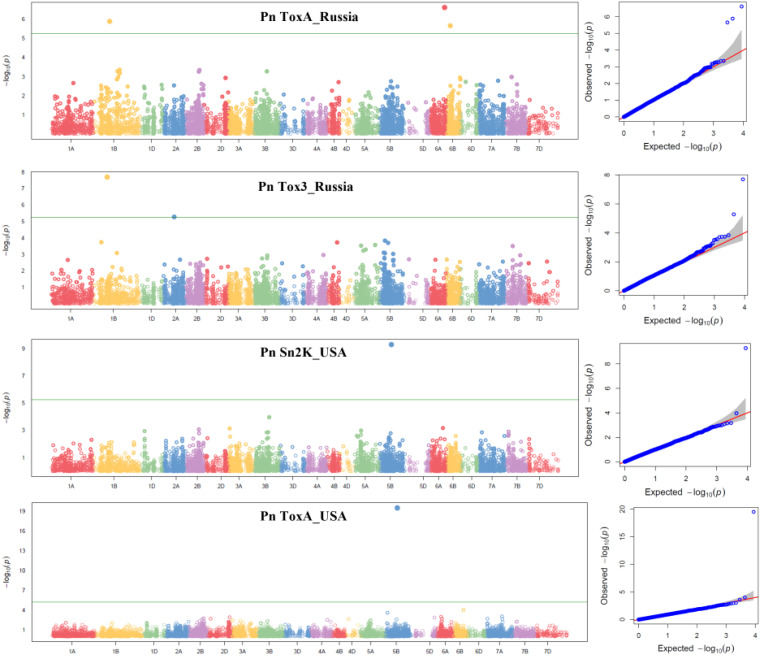
Manhattan and respective QQ plots for Pn ToxA_Russia, Pn Tox3_Russia, Pn Sn2K_USA, and Pn ToxA_USA in the GWAS panel phenotyped at greenhouse conditions during 2023. AUDPC, area under the disease progress curve; AUDPC 2020_Natural, 2020 screening in natural field conditions; AUDPC 2021_Artificial, 2021 screening in artificially inoculated field conditions.

Six Bonferroni-corrected MTAs including one pleiotropic MTA were detected for SNB and given in **Table 4** and represented in Manhattan plots in **Figure 3**. For the Pn ToxA_Russia isolate, three MTAs were identified. An MTA 100021621 was located on chromosome 6A mapped at 91.33 cM with the highest PVE of 17.00%, followed by MTA 1234457 with 16.68% PVE, located at 99.95 cM on chromosome 1B. The third MTA, 2275733, located at 23.41 cM on chromosome 6B explained 10.95% PVE. Two MTAs were identified for the Pn Tox3_Russia isolate. One MTA, 5971516, mapped at 86.77 cM on chromosome 1B explained 31.16% PVE and the second MTA, 1070935, mapped at 68.84 cM on chromosome 2A explained 13.70% PVE. One pleiotropic MTA, 100023665, mapped at 70.14 cM on 5B chromosome explained 46.74% PVE for the Pn ToxA_USA and 30.73% PVE for the Pn Sn2K_USA isolate.

**Table 4:** The list of MTAs identified for SNB in greenhouse and STB in field screening from the GWAS panel.

Traits	SNP	Chr.	Position (cM)	*P* value	Effect	PVE (%)
Greenhouse Experiments						
Pn ToxA_Russia	1234457	1B	99.95	1.29E−06	0.42	16.68
	100021621	6A	91.33	2.43E−07	−0.41	17.00
	2275733	6B	23.41	2.18E−06	−0.39	10.95
Pn Tox3_Russia	5971516	1B	86.77	1.99E−08	0.41	31.16
	1070935	2A	68.84	5.24E−06	0.29	13.70
Pn Sn2K_USA	100023665	5B	70.14	5.17E−10	−0.97	30.73
Pn ToxA_USA	100023665	5B	70.14	3.05E−20	−1.01	46.94
Field Studies						
AUDPC 2020_Natural	1230893	2B	78.31	1.22E−08	−39.72	22.90
	1202459	6B	79.15	1.34E−06	−33.47	12.21
AUDPC 2021_Artificial	1212480	3A	124.95	3.91E−06	−48.16	17.03

Pn ToxA_Russia, *P. nodorum* isolate 149-22_ToxA from Russia; Pn Tox3_Russia, *P. nodorum* isolate 118-22_Tox3 from Russia; Pn Sn2K_USA, *P. nodorum* isolate Sn2K from USA; Pn ToxA_USA, *P. nodorum* toxin SnToxA from USA; AUDPC, area under the disease progress curve; AUDPC 2020_Natural, 2020 screening in natural field conditions; AUDPC 2021_Artificial, 2021 screening in artificially inoculated field conditions.

Pn ToxA_Russia, *P. nodorum* isolate 149-22_ToxA from Russia; Pn Tox3_Russia, *P. nodorum* isolate 118-22_Tox3 from Russia; Pn Sn2K_USA, *P. nodorum* isolate Sn2K from USA; and Pn ToxA_USA, *P. nodorum* toxin SnToxA from USA.

#### 3.4.2 Field studies (natural and artificial infectious conditions)

**Figure 4:**
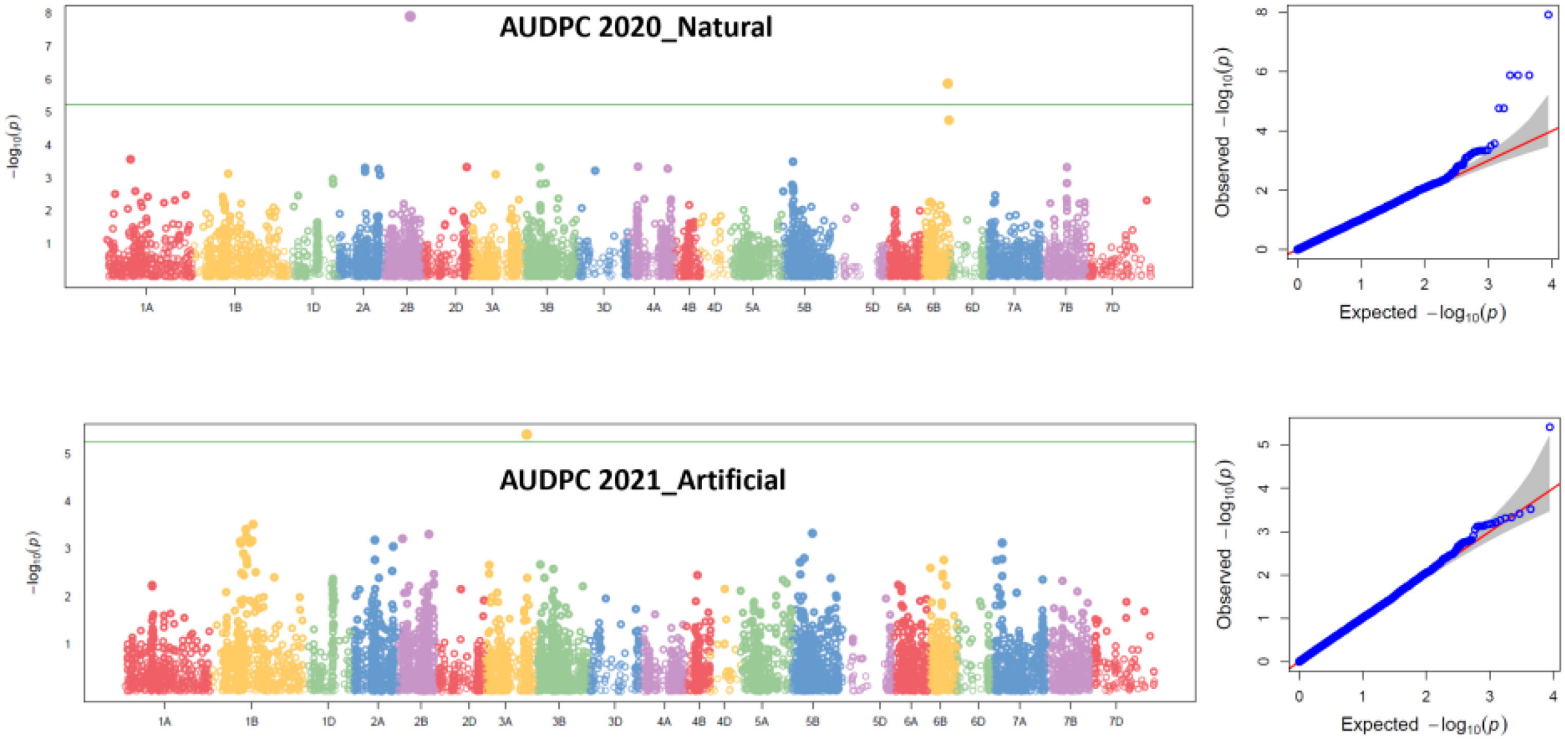
Manhattan and QQ plots for MTAs identified in 2020 natural field infection and 2021 artificial field infectious conditions in the GWAS panel. Pn ToxA_Russia, *P. nodorum* isolate 149-22_ToxA from Russia; Pn Tox3_Russia, *P. nodorum* isolate 118-22_Tox3 from Russia; Pn Sn2K_USA, *P. nodorum* isolate Sn2K from USA; and Pn ToxA_USA, *P. nodorum* toxin SnToxA from USA.

Two MTAs were identified for AUDPC scores under natural conditions during 2020. The MTA 1230893 mapped at 78.31 cM on 2B explained 22.90% PVE; similarly, the second MTA, 1202459, mapped at 79.15 cM on 6B chromosome explained 12.21% PVE. One MTA, 1212480, mapped at 124.95 cM on 3A chromosome explained 17.03% PVE under artificial infection for AUDPC scores during 2021 (**Table 4**, **Figure 4**).

**Figure 5:**
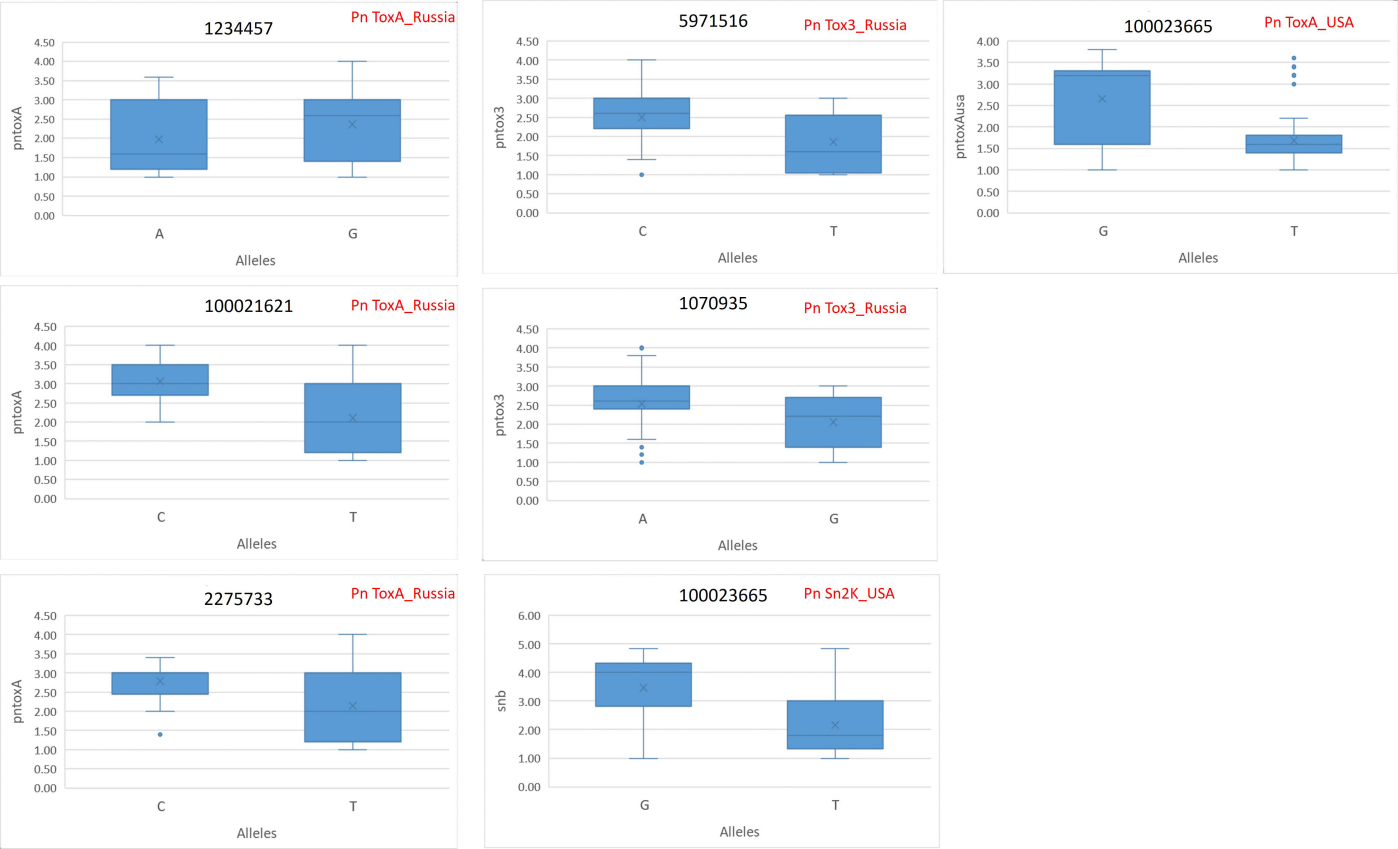
Phenotypic differences between favorable and unfavorable alleles of the MTAs identified for Pn ToxA_Russia, Pn Tox3_Russia, Pn Sn2K_USA, and Pn ToxA_USA in the GWAS panel phenotyped at greenhouse conditions during 2023. AUDPC, area under the disease progress curve; AUDPC 2020_Natural, 2020 screening in natural field conditions; AUDPC 2021_Artificial, 2021 screening in artificially inoculated field conditions.

**Figure 6:**
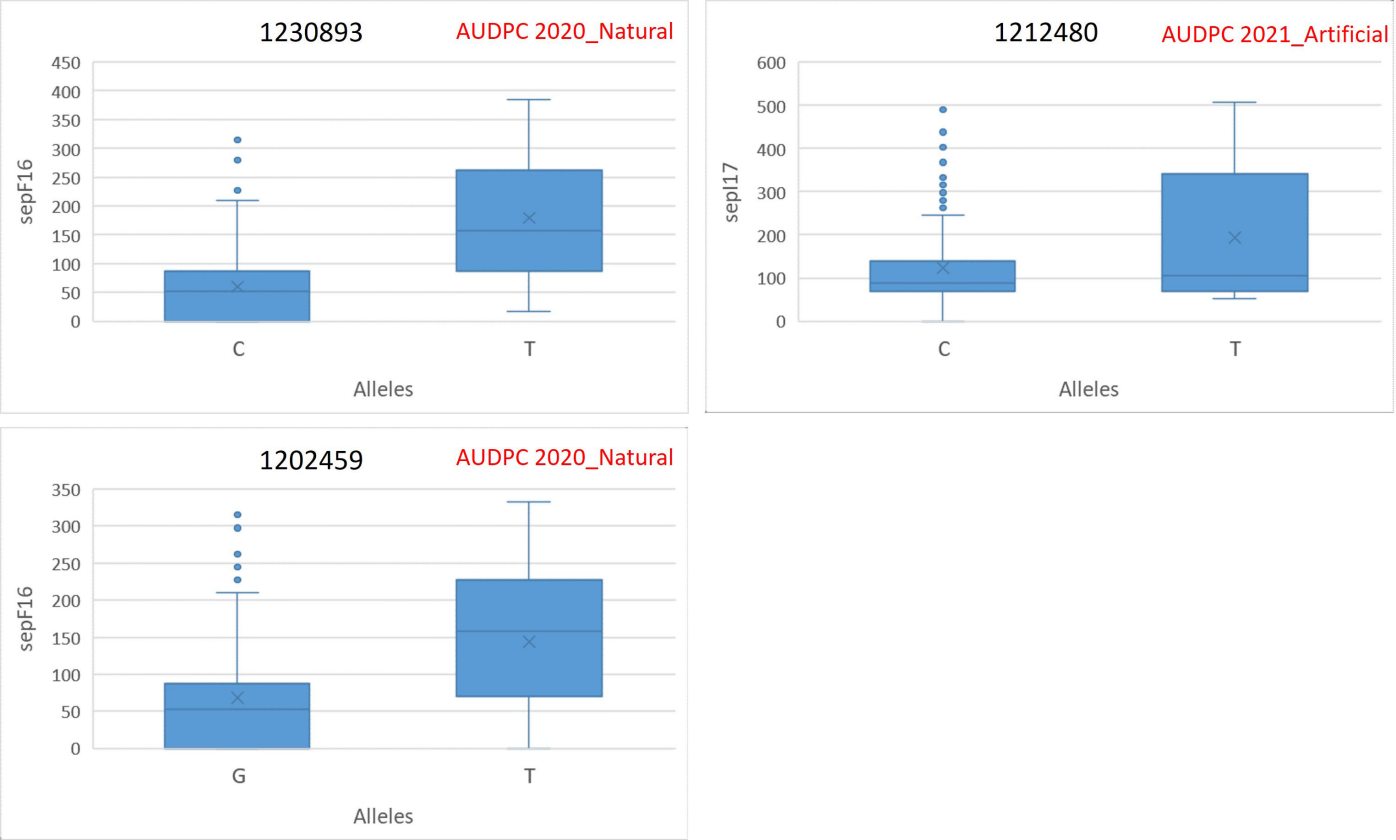
Phenotypic differences between favorable and unfavorable alleles of the MTAs identified in 2020 natural field infection and 2021 artificial field infection conditions in the GWAS panel.

The allelic differences between favorable and unfavorable alleles of the identified MTAs are depicted in boxplots. The boxplots for SNB resistance in GH are provided in **Figure 5**, and that for STB resistance in natural and artificial field infectious conditions is given in **Figure 6**. The percent difference between alleles for disease incidence is provided in **Supplementary Table S4**. There were three MTAs identified for Pn ToxA_Russia. The favorable allele for MTA 1234457 is A and that for 100021621 and 2275733 is T. The favorable allele of MTAs 1234457, 100021621, and 2275733 decreased the SNB incidence by 16.88%, 31.37%, and 23.30%, respectively. Two MTAs were identified for Pn Tox3_Russia with T and G as favorable alleles for MTAs 5971516 and 1070935, respectively. The favorable allele of MTAs 5971516 and 1070935 decreased the SNB disease by 25.60% and 18.97%, respectively. The pleiotropic MTA 100023665 for Pn Sn2K_USA and Pn ToxA_USA had T allele as the favorable allele. The favorable allele decreased the SNB incidence by 37.57% and 36.60% for Pn Sn2K_USA and Pn ToxA_USA, respectively. Additionally, two MTAs were identified for AUDPC 2020_Natural, and the favorable allele for MTA 1230893 is C and that for MTA 1202459 is G. The favorable allele of MTAs 1230893 and 1202459 decreased STB disease incidence by 66.53% and 52.37%, respectively. Finally, the MTA 1212480 identified for AUDPC 2021_Artificial had C as the favorable allele and the favorable allele decreased STB incidence by 36.13%.

### 3.5 *In silico* analysis

The SNPs linked to STB and SNB resistance were further used to identify the putative genes using the annotated wheat reference sequence (IWGSC RefSeq v2.1) and are given in **Table 5**. The genes falling in the 100-kb region flanking on either side of the marker were used to identify putative candidate genes. The region of MTA 100021621 associated with the Pn ToxA_Russia isolate possibly encodes a winged helix DNA-binding domain superfamily, an F-box-like domain superfamily, and an LRR domain superfamily. Similarly, an MTA, 2275733, associated with the Pn ToxA_Russia isolate encodes a protein response to low-sulfur, glycine–arginine–phenylalanine (GRF)-type zinc fingers (GRF-ZFs). An SNP 1234457 linked with Pn ToxA_Russia encodes Cytochrome P450. Another MTA, 5971516, for the Pn Tox3_Russia isolate encodes an RNA-binding S4 domain superfamily. Similarly, 1070935 associated with the Pn Tox3_Russia isolate encodes the haem peroxidase superfamily, peroxidases haem-ligand binding site pleiotropic MTA 100023665 associated with Pn Sn2K_USA and Pn ToxA_USA isolates, and encodes potassium transporter, palmitoyltransferase, nucleotide-binding alpha-beta plait domain superfamily, and RNA-binding domain superfamily. SNP 1230893 for AUDPC 2020_Natural encodes the DNA-binding domain superfamily, disease resistance protein, NB-ARC, LRR domain superfamily, P-loop containing nucleoside triphosphate hydrolase, virus X resistance protein, O-methyltransferase domain, and S-adenosyl-L-methionine-dependent methyltransferase superfamily. Similarly, 1202459 encodes for the LRR domain superfamily, NB-ARC, and virus X resistance protein. For AUDPC 2021_Artificial, SNP 1212480 on chromosome 3A encodes protein of unknown function DUF247.

**Table 5:** Putative candidate genes in the region of STB and SNB tolerance linked MTAs.

Trait	SNP	Chr	GP (cM)	PP (Mb)	TraesID	Putative candidate gene
Pn ToxA_Russia	1234457	1B	99.95	190.7	TraesCS1B03G0380700.1	Cytochrome P450
	100021621	6A	91.33	607.9	TraesCS6A03G0980600.1	S-adenosyl-L-methionine-dependent methyltransferase superfamily, Winged helix DNA-binding domain superfamily
					TraesCS6A03G0981000.1	F-box-like domain superfamily, Leucine-rich repeat (LRR) domain superfamily
					TraesCS6A03G0981200.1	Domain of unknown function DUF3741 and DUF4378
	2275733	6B	23.41	112.8	TraesCS6B03G0289700.1	Protein response to low sulfur
					TraesCS6B03G0289600.1	Glycine-arginine-phenylalanine (GRF)-type zinc fingers (GRF-ZFs)
Pn Tox3_Russia	5971516	1B	86.77	148.8	TraesCS1B03G0326800.1	RNA-binding S4 domain superfamily
	1070935	2A	68.84	529.7	TraesCS2A03G0760700.1	Haem peroxidase superfamily, Peroxidases haem-ligand binding site
					TraesCS2A03G0760900.1	Mog1/PsbP, alpha/beta/alpha sandwich, PsbP, C-terminal
Pn Sn2K_USA & Pn ToxA_USA	100023665	5B	70.14	549.9	TraesCS5B03G0923200.1	Potassium transporter
					TraesCS5B03G0923300.1	Palmitoyltransferase
					TraesCS5B03G0922900.1	Nucleotide-binding alpha-beta plait domain superfamily, RNA-binding domain superfamily
AUDPC 2020_Natural	1230893	2B	78.31	605.1	TraesCS2B03G1063600.1	AP2/ERF domain superfamily, DNA-binding domain superfamily
	1202459	6B	79.15	718.2	TraesCS6B03G1249200.1	Disease resistance protein, NB-ARC, LRR domain superfamily, P-loop containing nucleoside triphosphate hydrolase, Winged helix-like DNA-binding domain superfamily, Virus X resistance protein
					TraesCS6B03G1248900.1	O-methyltransferase domain, S-adenosyl-L-methionine-dependent methyltransferase superfamily, Winged helix-like DNA-binding domain superfamily
AUDPC 2021_Artificial	1212480	3A	124.95	721.1	TraesCS3A03G1160000.1	Protein of unknown function DUF247

Pn ToxA_Russia, *P. nodorum* isolate 149-22_ToxA from Russia; Pn Tox3_Russia, *P. nodorum* isolate 118-22_Tox3 from Russia; Pn Sn2K_USA, *P. nodorum* isolate Sn2K from USA; Pn ToxA_USA, *P. nodorum* toxin SnToxA from USA; AUDPC, area under the disease progress curve; AUDPC 2020_Natural, 2020 screening in natural field conditions; AUDPC 2021_Artificial, 2021 screening in artificially inoculated field conditions; GP, genetic position; PP, physical position.

## 4 Discussion

STB and SNB caused by *Z. tritici* and *P. nodorum*, respectively, are two important biotic threats to wheat production globally. Therefore, breeding for genetically resistant cultivars is the ideal approach for obtaining sustainable yields. Although it is important to provide major gene-based resistance by introducing new genes, polygene-based resistance governed by QTLs is important when major genes fail. LD decay is important in GWASs because it determines the density of genetic markers needed to accurately identify associated loci, as the rate at which LD diminishes across the genome suggests how closely spaced markers must be to effectively pinpoint causal genes within a region of interest; essentially, a faster LD decay indicates that a higher marker density is required to capture the markers close enough to the causal loci. In the present study, LD decay was much slower in the D subgenome as compared to the A and B subgenomes. Previous studies reported both a slower rate of LD decay (Wang et al., 2014) and a faster rate of LD decay in subgenome D, followed by subgenomes A and B (Voss-Fels et al., 2015). The variations in LD decay patterns among the subgenomes may be attributed to variations in the study materials, levels of gene flow, population stratifications, and the degree of selection pressure (Mazumder et al., 2024). The other key reason for slow LD decay of the D subgenome is due to its late introduction to make a hexaploid wheat from tetraploid wheat during domestication.

In the current study, nine Bonferroni-corrected MTAs including one pleiotropic MTA were detected for resistance to STB and SNB. Of the identified nine MTAs, six were race-specific MTAs for SNB identified in greenhouse conditions and three were non-race-specific MTAs (two MTAs under natural infectious field conditions and one MTA under artificial infectious field conditions) for STB resistance identified in field conditions. The MTA 100021621 located on chromosome 6A for the Pn ToxA_Russia isolate was identified at 91.33 cM. Previously, Francki et al. (2020) detected an MTA on chromosome 6A at 89.33 cM with a PVE of 9% for mixed isolates of *P. nodorum*; in the same study, two more MTAs on the same chromosome were identified at 61.42 and 333.95 cM with a PVE of 10% and 8%, respectively. Hence, the location of the MTA 100021621 identified in the present study at 91.33 cM was similar to the previously identified MTA at 89.33 cM on the same 6A chromosome. The second race-specific (Pn ToxA_Russia isolate) MTA 1234457 was mapped at 99.95 cM on chromosome 1B. Similarly, a third race-specific (Pn Tox3_Russia isolate) MTA, 5971516, was mapped at 86.77 cM on chromosome 1B. Ac similar race-specific MTA, 1129298, for SNB was reported in the previous study of Navathe et al. (2023), and they identified MTA at 450.5 cM on chromosome 1B. The fourth race-specific (Pn ToxA_Russia isolate) MTA, 2275733, mapped at 23.41 cM on chromosome 6B with a PVE of 10.95%. Previously, a QTL (*QSnb.nmbu-6BL*) was identified at 718–721 Mb on chromosome 6B and *QSnb.nmbu-2AS* at 4–24 Mb on chromosome 2A by Lin et al. (2022). Similarly, Navathe et al. (2023) identified an MTA, 1085698, at 66.91 cM on the 6B chromosome and another MTA, 1094287, at 88.18 cM on chromosome 2A.

One important pleiotropic MTA, 100023665, mapped at 70.14 cM on chromosome 5B was identified for the two isolates (Pn ToxA_USA isolate and Pn Sn2K_USA). A previous study by Phan et al. (2018) identified MTA 1168841 for SnToxA on the 5B chromosome at 137.096 cM. In the same study, three MTAs were identified at 7.025 cM for SnTox1 on the 1B chromosome and an MTA, 1151694, was identified for two different SNB isolates SnTox3 and SN15 SNB at 5.452 cM on the 5B chromosome. Similarly, two MTAs were identified between 350–370 Mb and 662–668 Mb (Lin et al., 2022), and one MTA was identified between 546 and 547 Mb (Friesen et al., 2006) on the same 5B chromosome. Singh et al. (2019) identified two MTAs between marker intervals of *wPt-3661–wPt-3457* and *XFCP393–wPt-1733* at 168 and 180 cM, respectively, on the 5B chromosome with 8% and 19.51% PVE. Race-specific QTLs are very important because plant breeders can practice a targeted breeding strategy to identify genomic regions that confer resistance to particular isolates of the SNB pathogen *P. nodorum*; this will enable the development of more targeted and effective resistant wheat cultivars through marker-assisted gene pyramiding to prevalent pathogen strains in a particular geographic area to prevent the crop yield losses. Therefore, the pleiotropic MTA 100023665 identified in the present study on the 5B chromosome is a potential putative candidate in SNB resistance breeding in wheat, as several MTAs have been harbored on the same chromosome at different positions in the previous studies.

One MTA, 1230893, mapped at 78.31 cM on chromosome 2B explained 22.9% PVE for AUDPC scores under natural field infection conditions during 2020. Previously, Alemu et al. (2021) identified an MTA, *Kukri_rep_c103893_875*, on a 2B chromosome at 65 cM under natural infections for STB disease. Similarly, Mekonnen et al. (2021) reported two QTLs *qSTB.09* and *qSTB.10*, respectively, at 237.98 and 698.10 Mb with 6.63% and 9.84% PVE on 2B chromosome. Similarly, Kidane et al. (2017) reported a QTL *qSTB.2* at 85.8 cM on the 2B chromosome. The second MTA, 1202459, mapped at 79.15 cM on chromosome 6B with 12.21% PVE was identified for AUDPC scores under natural conditions during 2020. Previously, an SNP, *BS00048295_51*, was identified at five environments at 51.22, 104.92, 133.69, 134.69, and 141.88 cM with 7.7%–17.94% PVE on 6B chromosome under natural infection conditions for STB (Riaz et al., 2020). Also, Alemu et al. (2021) identified two MTAs at 76 and 113 cM on 6B chromosome under natural infections for STB disease. Similarly, Mekonnen et al. (2021) reported an MTA at 706.98 Mb with 6.13% to 9.91% PVE on the 6B chromosome. The third MTA, 1212480, mapped at 124.95 cM on 3A chromosome was identified under artificial infection for AUDPC scores during 2021. A previous study by Kidane et al. (2017) reported a QTL *qSTB.3* at 71.6–72.5 cM on the 3A chromosome. Similarly, Mekonnen et al. (2021) reported three MTAs at 8.74, 161.44, and 710.34 Mb with 9.82%, 2.92%, and 9.92% PVE on the 3A chromosome, respectively. Understanding the genetic basis of STB resistance under natural and artificial inoculation conditions through QTL mapping is essential to breed field tolerance varieties through marker-assisted breeding to reduce the STB associated crop damages in wheat.

The putative genes identified in the regions of the MTAs that were linked to disease resistance are presented in **Table 5**. For instance, an SNP, 1234457, on 1B chromosome associated with Pn ToxA_Russia encodes cytochrome P450 (TraesCS1B03G0380700.1). Cytochrome P450s (CYPs) are involved in plant defense and detoxification and host response to diseases, including the wheat response to Fusarium head blight (Walter et al., 2008; Walter and Doohan et al., 2011) and Septoria leaf blotch disease (Kay et al., 2024). Similarly, SNPs 100021621 and 1202459 encoding the LRR domain superfamily (TraesCS6A03G0981000.1 for PnToxA_Russia and TraesCS6B03G1249200.1 for AUDPC 2020 natural field infectious conditions) regulates disease resistance in plants. Similarly, SNPs 100021621 (TraesCS6A03G0980600.1), 5971516 (TraesCS1B03G0326800.1), 100023665 (TraesCS5B03G0922900.1), and 1202459 (TraesCS6B03G1248900.1) encode nucleotide-binding sites (NBSs). NBS-LRR (nucleotide-binding site–leucine-rich repeat) class proteins are an important class of pathogenesis-related proteins in plants. They get activated in response to pathogen effectors and cause hypersensitive response (HR) to inhibit the pathogen growth (Kang et al., 2016; Zhou et al., 2020). The sensitivity to ToxA is governed by the *Tsn1* gene present on 5BL in wheat. *Tsn1* was found to have disease resistance gene-like features, including S/TPK and NBS-LRR domains (Faris et al., 2010).

Another MTA, 1202459, for AUDPC 2020 natural field infectious conditions located on 6B chromosome at 79.15 cM encoded multiple proteins like disease resistance protein, NB-ARC, LRR domain superfamily, P-loop containing nucleoside triphosphate hydrolase, winged helix-like DNA-binding domain superfamily, and virus X resistance protein (TraesCS6B03G1249200.1) and regulates disease resistance in wheat. The NB-ARC–NPR1 fusion protein negatively regulates the defense response in wheat to stem rust pathogen (Wang et al., 2020). Furthermore, SNP 2275733 on the 6B chromosome encodes GRF-ZFs (TraesCS6B03G0289600.1). Zinc finger binding domains are present in the well-known plant resistance proteins NBS-LRRs that are involved in the effector-triggered immune response. Most importantly, a pleiotropic SNP 100023665 on 5B at 70.14 cM encodes potassium transporter, the nucleotide-binding alpha-beta plait domain superfamily, and the RNA-binding domain superfamily associated with the resistance to isolate Pn Sn2K_USA and toxin Pn ToxA_USA. Previously, Khan et al. (2024); Navathe et al. (2023); Kumar et al. (2022); Phan et al. (2021), and Gupta et al. (2012) also reported candidate genes NBS-LRR, zinc finger, and potassium transporter through *in silico* analysis in wheat through GWASs.

## 5 Conclusion

Most wheat-growing regions are experiencing recurrent epidemics caused by biotic stresses including Septoria blotch (SNB and STB). This can lead to sizeable yield losses and affect grain quality. The present study has identified nine MTAs for resistance to SNB and STB under both greenhouse and field conditions (natural and artificial infections) along with the candidate genes, which will prove valuable to enhance Septoria resistance in wheat. The pleiotropic MTA (100023665) with 30.73% and 46.94% PVE was associated with important putative candidate genes such as the NBS domain superfamily, the members of which are known to confer plant defense responses. Few other MTAs associated with disease resistance protein, the LRR domain superfamily, and zinc finger GRF type are also useful candidates. The identified MTAs, particularly MTAs with high PVE and pleiotropic MTA, could be utilized for marker-assisted breeding after validation. The functional characterization of the candidate genes will provide insights into the genetic basis of Septoria resistance.

## Data Availability

The data presented in the study are deposited in the DRYAD repository, with DOI: 10.5061/dryad.8gtht7711.
